# The complexity of frugal colouring

**DOI:** 10.1007/s40065-021-00311-7

**Published:** 2021-01-29

**Authors:** Stefan Bard, Gary MacGillivray, Shayla Redlin

**Affiliations:** 1 Department of Mathematics and Statistics, University of Victoria, Victoria, BC Canada; 2 Department of Combinatorics and Optimization, University of Waterloo, Waterloo, ON Canada

**Keywords:** 05C15, 68Q17

## Abstract

A *t*-*frugal colouring* of a graph *G* is an assignment of colours to the vertices of *G*, such that each colour appears at most *t* times in the neighbourhood of any vertex. A dichotomy theorem for the complexity of deciding whether a graph has a 1-frugal colouring with *k* colours was found by McCormick and Thomas, and then later extended to restricted graph classes by Kratochvil and Siggers. We generalize the McCormick and Thomas theorem by proving a dichotomy theorem for the complexity of deciding whether a graph has a *t*-frugal colouring with *k* colours, for all pairs of positive integers *t* and *k*. We also generalize bounds of Lih et al. for the number of colours needed in a 1-frugal colouring of a given $K_4$-minor-free graph with maximum degree $\Delta$ to *t*-frugal colourings, for any positive integer *t*.

## Introduction and background

A colouring of a graph *G* is *t -frugal* if, for every $v \in V$ and for every colour *c*, the colour *c* is assigned to at most *t* vertices of *N*(*v*). We consider only proper colourings.

Frugal colourings were introduced by Hind, Molloy, and Reed in 1997 [16]. Their main result is that every graph with sufficiently large maximum degree $\Delta$ has a $\lceil \log ^8 \Delta \rceil$-frugal $(\Delta + 1)$-colouring. Molloy and Reed later proved that every graph *G* with sufficiently large maximum degree has a $(50\log \Delta / \log \log \Delta )$-frugal $(\Delta +1)$-colouring [22]. Their work was generalized by Kang and Müller [18].

Rather than focussing on the values of *t* for which a graph has a *t*-frugal colouring with a given number of colours, we fix *t* and focus on the number of colours needed for a *t*-frugal colouring. The *t -frugal chromatic number* of *G* is the smallest positive integer *k*, such that there exists a *t*-frugal *k*-colouring of *G*, and is denoted by $\chi _t(G)$.

Amini, Esperet, and van den Heuvel [1] examined *t*-frugal colourings of planar graphs and outerplanar graphs. For planar graphs, they showed $\chi _t \le \left\lfloor \frac{2\Delta + 19}{t} \right\rfloor$, and gave improvements for planar graphs of sufficiently large girth and maximum degree. For outerplanar graphs with maximum degree $\Delta \ge 3$, they showed $\chi _t \le \left\lfloor \frac{\Delta - 1}{t} \right\rfloor + 3$ for $t \ge 2$. The bound was improved for 2-connected outerplanar graphs with maximum degree at least 7.

The case $t=1$ has a substantial history under the names *injective colouring*, *distance 2 colouring*, *colouring the square*, and *L*(1, 1)*-labelling*; for example, see [5, 6, 11, 12, 26]. These colourings arise as an example in the monadic second-order logic of graphs [7]. Similar concepts were considered earlier; see [17], pages 156-158. The smallest number of colours needed in a 1-frugal colouring of a graph *G* is $\chi (G^2)$, where $G^2$ is the graph obtained from *G* by adding edges between all pairs of vertices at distance 2 in *G*.

Wegner [26] conjectured that if *G* is planar, then:

$$\begin{aligned} \chi _1(G) \le {\left\{ \begin{array}{ll} 7 \quad \text{ if }\ \Delta = 3,\\ \Delta + 5\quad \text{ if }\ 4 \le \Delta \le 7,\\ \left\lfloor \frac{3\Delta }{2} \right\rfloor + 1\quad \text{ if }\ \Delta \ge 8.\\ \end{array}\right. } \end{aligned}$$

The case $\Delta = 3$ has been settled [13, 25]. In the case $\Delta \ge 8$, Molloy and Salavatipour [23] established the bound $\chi _1(G) \le \left\lfloor \frac{5}{3} \Delta \right\rfloor + 78$, and showed that the additive constant can be replaced by 25 for large $\Delta$. Havet, van den Heuvel, McDiarmid, and Reed [14] later showed that the (list) chromatic number of the square of a planar graph with $\Delta \ge 8$ is at most $\frac{3}{2}\Delta (1 + o(1))$. Lih et al. [21] proved a restricted version of the conjecture by showing that if *G* is a $K_4$-minor-free graph, then:

$$\begin{aligned} \chi _1(G) \le {\left\{ \begin{array}{ll} \Delta + 3\quad \text{ if }\ 2 \le \Delta \le 3,\\ \left\lfloor \frac{3\Delta }{2} \right\rfloor + 1\quad \text{ if }\ \Delta \ge 4.\\ \end{array}\right. } \end{aligned}$$

We generalize this result to all $t \ge 1$ by proving:

### Theorem 1.1

Let *G* be a $K_4$-minor-free graph and $t \ge 1$. Then:

$$\begin{aligned} \chi _t(G) \le {\left\{ \begin{array}{ll} 3 + \left\lfloor \frac{\Delta - 1}{t} \right\rfloor + \left\lfloor \frac{1}{t} \right\rfloor &{} 2 \le \Delta \le 3 \\ 3 + \left\lfloor \frac{\left\lfloor \frac{3\Delta }{2} \right\rfloor - 2}{t} \right\rfloor &{} \Delta \ge 4. \end{array}\right. } \end{aligned}$$

The proof of this theorem appears in Sect. 2.

McCormick and Thomas [24] determined the complexity of deciding whether a given graph has a 1-frugal *k*-colouring. Their result was later strengthened by Kratochvíl and Siggers [20]. The problem is NP-complete for every fixed $k \ge 4$ when the input is restricted to planar graphs. The case $k = 4$ follows from [10]. The problem is polynomially solvable for each $k \le 3$. For example, a graph has a 1-frugal 3-colouring if and only if every component is either a path, or a cycle of length divisible by 3. It is known that every planar graph with $\Delta \le \frac{5}{3}k - 52$ has a 1-frugal *k*-colouring [23]. Kratochvíl and Siggers also consider degree restrictions and show that, for each $k \ge 7$, the problem of deciding whether a given graph with maximum degree at most $2\sqrt{k-1}$ has a 1-frugal *k*-colouring is NP-complete. By contrast, it follows from Brooks’ Theorem (applied to the square) that any graph with maximum degree at most $\sqrt{k-1}$ has a 1-frugal *k*-colouring.

We generalize the theorem of McCormick and Thomas [24] to all pairs of positive integers *t* and *k* by proving:

### Theorem 1.2

If $k \le 2$, or $k = 3$ and $t = 1$, then the problem of deciding whether a given graph has a *t*-frugal *k*-colouring is solvable in polynomial time. If $k = 3$ and $t \ge 2$, or $k \ge 4$ and $t \ge 1$, then the problem of deciding whether a given graph has a *t*-frugal *k*-colouring is NP-complete.

The proof of this theorem appears in Sect. 3.

For all integers *t* and *k*, the existence of a *t*-frugal *k*-colouring can be described in monadic second-order logic. Hence, by Courcelle’s Theorem [7], the existence of such a colouring can be decided in linear time for graphs of bounded treewidth.

We conclude this section by briefly mentioning some related work. Locally injective colourings which need not be proper colourings are considered, for example, in [4, 9, 12, 15, 20, 28]. For recent work on locally injective colourings of digraphs, see [3]. Colourings of a graph in which all vertices at distance at most *d* must be assigned different colours are discussed in [19, 27]. Results on *L*(*i*, *j*)-labellings, which generalize *L*(1, 1)-labellings, are surveyed in [5, 6]

## $K_4$-minor-free graphs

A graph *G* is called $K_4$-minor-free if no subgraph of *G* can be transformed into $K_4$ by contracting edges. The $K_4$-minor-free graphs are exactly the graphs of treewidth 2. This graph class properly contains the outerplanar graphs.

As noted in the previous section, the existence of a *t*-frugal *k*-colouring can be decided in linear time for graphs of bounded treewidth, hence in particular for $K_4$-minor-free graphs. The decision algorithm can be combined with binary search to obtain a $O(n \log n)$ algorithm to compute the *t*-frugal chromatic number of a $K_4$-minor-free graph with *n* vertices. However, since $K_4$-minor-free graphs have bounded clique-width, the *t*-frugal chromatic number can be computed in linear time [2, 8].

In this section, we prove Theorem 1.1, which generalizes the theorem of Lih et al. [21]. Our approach is similar to theirs.

Let *G* be a $K_4$-minor-free graph, and let *u* be a vertex of *G*. Let $S(u) = \{x | d(x) \ge 3 \text { and either } xu \in E \text { or there exists } w, d(w) = 2 \text { s.t. } xw, uw \in E\}$, and let $D(u) = |S(u)|$. We make use of the following structural lemma.

### Lemma 2.1

[21] Let *G* be a $K_4$-minor-free graph. Then, one of the following holds:

- $$\delta (G) \le 1$$;
- there exist two adjacent vertices of degree 2;
- there exists a vertex *u* with $d(u) \ge 3$ and $D(u) \le 2$.

Using this lemma, we now prove Theorem 1.1.

### *Proof *(Theorem 1.1)

The case $t=1$ is the theorem of Lih et al. [21]. Hence, assume $t \ge 2$. Note that $\left\lfloor \frac{1}{t} \right\rfloor = 0$.

If $\Delta = 2$, then any proper colouring of *G* is a proper *t*-frugal colouring of *G*. Since $K_4$-minor-free graphs have chromatic number at most 3, and $3 + \left\lfloor \frac{\Delta - 1}{t} \right\rfloor = 3$ when $\Delta = 2$ and $t \ge 2$, the theorem holds for $\Delta = 2$. Hence, assume $\Delta \ge 3$.

For convenience, let:

$$\begin{aligned} K(\Delta ) = {\left\{ \begin{array}{ll} 3 + \left\lfloor \frac{\Delta - 1}{t} \right\rfloor \quad \text{ if }\ \Delta = 3, \\ 3 + \left\lfloor \frac{\left\lfloor \frac{3\Delta }{2} \right\rfloor - 2}{t} \right\rfloor \quad \text{ if }\ \Delta \ge 4. \end{array}\right. } \end{aligned}$$

The theorem is clearly true for graphs with at most 4 vertices. Hence, assume $|V(G)| \ge 5$.

We colour *G* by induction on $n = |V(G)|$. The base cases $1 \le n \le 4$ are covered above. Suppose the statement holds for all graphs on at most $n-1$ vertices. Let *G* be a $K_4$-minor-free graph on *n* vertices. Our strategy is to delete a vertex *w* to obtain $H = G - w$. The graph *H* can be coloured by induction. We then seek to extend the colouring of *H* to *G* by assigning a colour to *w*. We will show that the number of colours which are excluded as options for the colour of *w* is less than the number of colours which are available; hence, an extension exists. For a vertex *w* in *G*, let $f_t(w)$ be the maximum number of colours which cannot be used to extend a proper *t*-frugal colouring of *H* to a proper *t*-frugal colouring of *G*. Colours are excluded if they are used to colour a vertex adjacent to *w*, or are used *t* times in the neighbourhood of some neighbour of *w*. Therefore, $f_t(w) \le |N(w)| + \left\lfloor \frac{|N_2(w)|}{t} \right\rfloor$, where $N_2(w)$ is the set of vertices at distance 2 from *w* in *G*.

Suppose first that $\delta = 1$. Let *w* be a vertex with degree 1. Clearly, $H = G-w$ is $K_4$-minor-free and $\Delta (H) \le \Delta (G)$. Therefore, *H* can be *t*-frugally coloured with $K(\Delta )$ colours. Since $f_t(w) \le |N(w)| + \left\lfloor \frac{|N_2(w)|}{t} \right\rfloor \le 1 + \left\lfloor \frac{\Delta - 1}{t} \right\rfloor < K(\Delta )$. Therefore, a *t*-frugal colouring of *H* can be extended to a *t*-frugal colouring of *G*.

Next, suppose that there are vertices *v* and *w*, such that $d(v) = d(w) = 2$ and $vw \in E$. Consider $H = G - w$. By assumption, *H* can be coloured using $K(\Delta )$ colours. We know that *w* and *v* have degree 2, and $t \ge 2$, no valid choice of colour for *w* can violate the frugality condition for *v*. Therefore, the neighbour of *v* at distance 2 from *w* can be disregarded which leads to the slightly improved bound $f_t(w) \le |N(w)| + \left\lfloor \frac{|N_2(w) - 1|}{t} \right\rfloor \le 2 + \left\lfloor \frac{(\Delta - 1)}{t} \right\rfloor < K(\Delta )$. The colouring of *H* can therefore be extended to a *t*-frugal colouring of *G*.

By Lemma 2.1, if $\delta = 2$ and the previous case does not apply (i.e., any two vertices which have degree two are non-adjacent), then there is a vertex *u*, such that $d(u) \ge 3$ and $D(u) \le 2$. Furthermore, since $\delta \ge 2$, it follows that $D(u) \ge 1$. Therefore, $1 \le D(u) \le 2$. For a vertex $x \in S(u)$, let $M_x$ be the set of degree two vertices which are adjacent to both *u* and *x*, and let $m_x = |M_x|$.

Suppose that $D(u) = 1$, and let $S(u) = \{x\}$. Then, the neighbourhood of *x* contains the neighbourhood of *u*, because no two vertices of degree two are adjacent and *x* is the only possible neighbour of *u* which does not have degree two. (Note that *u* and *x* are not necessarily adjacent.) Since $d(u) \ge 3$, we have $m_x \ge 2$. Let $w \in M_x$ and $H = G - w$, and we may colour *H* with $K(\Delta )$ colours as before. Since the neighbourhood of *x* contains the neighbourhood of *u*, we have $f_t(w) \le |N(w)| + \left\lfloor \frac{|N_2(w)|}{t} \right\rfloor \le 2 + \left\lfloor \frac{(\Delta - 1)}{t} \right\rfloor < K(\Delta )$. The colouring of *H* can therefore be extended to a *t*-frugal colouring of *G*.

Suppose that $D(u) = 2$, and let $S(u) = \{x,y\}$. Without loss of generality, assume $m_x \ge m_y$. Since $d(u) \ge 3$, we have $m_x \ge 1$. Let $w \in M_x$ and $H = G - w$. Suppose *H* is *t*-frugally coloured using $K(\Delta )$ colours. We consider cases based on the possible adjacencies of *x* and *y* to *u*.

First, suppose that $xu \in E$. Since $D(u) = 2$, we have $m_x + m_y \ge d(u) - 2$, and since $m_x \ge m_y$, we have $m_x \ge \left\lceil \frac{d(u)-2}{2} \right\rceil = \left\lceil \frac{d(u)}{2} \right\rceil - 1$. Therefore:

$$\begin{aligned} f_t(w)&\le |N(w)| + \left\lfloor \frac{|N_2(w)|}{t} \right\rfloor \\&= 2 + \left\lfloor \frac{d(u) + d(x) - m_x - 3}{t} \right\rfloor \\&\le 2 + \left\lfloor \frac{\Delta + \Delta - (\left\lceil \frac{\Delta }{2} \right\rceil - 1) - 3}{t} \right\rfloor \\&= 2 + \left\lfloor \frac{2\Delta - \left\lceil \frac{\Delta }{2} \right\rceil - 2}{t} \right\rfloor \\&= 2 + \left\lfloor \frac{\left\lfloor \frac{3\Delta }{2} \right\rfloor - 2}{t} \right\rfloor < K(\Delta ). \end{aligned}$$

The colouring of *H* can therefore be extended to a *t*-frugal colouring of *G*.

Now, suppose that $xu \not \in E$ and $yu \not \in E$. In this case, we have $m_x + m_y = d(u)$, so $m_x \ge \left\lceil \frac{d(u)}{2} \right\rceil$. Let *c* be a *t*-frugal colouring of $H = G - w$ with $K(\Delta )$ colours.

If *u* is not adjacent to a vertex with colour *c*(*x*), then either $c(u) = c(x)$ or *u* can be recoloured, so that $c(u) = c(x)$. It then follows that:

$$\begin{aligned} f_t(w)&\le (|N(w)| - 1) + \left\lfloor \frac{|N_2(w)|}{t} \right\rfloor \\&= 1 + \left\lfloor \frac{d(u) + d(x) - m_x - 1}{t} \right\rfloor \\&\le 1 + \left\lfloor \frac{\Delta + \Delta - \left\lceil \frac{\Delta }{2} \right\rceil - 1}{t} \right\rfloor \\&= 1 + \left\lfloor \frac{\left\lfloor \frac{3\Delta }{2} \right\rfloor - 1}{t} \right\rfloor \\&\le 2 + \left\lfloor \frac{\left\lfloor \frac{3\Delta }{2} \right\rfloor - 2}{t} \right\rfloor < K(\Delta ). \end{aligned}$$

The colouring *c* can therefore be extended to a *t*-frugal colouring of *G*.

If *u* is adjacent to a vertex with colour *c*(*x*), then we have:

$$\begin{aligned} f_t(w)&\le |N(w)|+ \left\lfloor \frac{|N_2(w)| - 1}{t} \right\rfloor \\&\le 2 + \left\lfloor \frac{\left\lfloor \frac{3\Delta }{2} \right\rfloor - 2}{t} \right\rfloor < K(\Delta ) . \end{aligned}$$

The colouring *c* can therefore be extended to a *t*-frugal colouring of *G*.

Finally, suppose that $xu \not \in E$ and $yu \in E$. In this case, $m_x + m_y = d(u) - 1$. If $m_x = m_y$, we may use the method from the case $xu \in E$. Hence, assume that $m_x > m_y$. Then, $m_x \ge \left\lceil \frac{d(u)}{2} \right\rceil$, and we may then proceed as in the previous case.

This completes the proof. $\square$

## Complexity of proper frugal colouring

The decision problem *P*(*t*, *k*)-*colouring* is formally defined as follows.

**Problem:** *P*(*t*, *k*)-colouring, $$t \ge 1$$, $$k \ge 1$$.

**Instance:** A graph *G*.

**Question:** Is there a proper *t*-frugal *k*-colouring of *G*?

*P*(*t*, *k*)-colouring clearly belongs to NP for all integers $t \ge 1$ and $k \ge 1$.

NP-completeness of *P*(1, *k*)-colouring for each $k \ge 4$ follows from results of McCormick and Thomas [24] (also see Kratochvil and Siggers [20]).

We will extend the result to *P*(*t*, *k*)-colouring, for all pairs of positive integers *t* and *k*. We first treat some special cases, and then derive the general result from them.

### Lemma 3.1

*P*(2, 3)-colouring is NP-complete.

### Proof

The transformation is from 3-colouring.

**Fig. 1:**
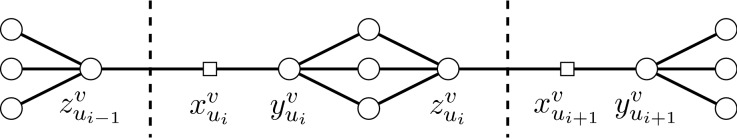
A portion of the vertex-gadget $F^v$, where $H^v_{u_i}$ is the graph shown between the dotted lines

Let *G* be an instance of 3-colouring. We construct an instance $G'$ of *P*(2, 3)-colouring from *G* as follows. First, consider the graph *H* with vertices *x*, *y*, and *z*, such that *x* is adjacent to *y*, and the vertices *y* and *z* are connected by three paths of length two. For each $v \in V(G)$, we construct a vertex-gadget $F^v$ from copies of *H* as follows. For each vertex *u*, such that $uv \in E(G)$, create a copy $H_u^v$ of *H* in which the vertices $x_u^v$, $y_u^v$, and $z_u^v$ correspond to the vertices *x*, *y*, and *z* of *H*, respectively. Let $N(v) = \{u_1,u_2,\ldots ,u_{\delta (v)}\}$. For $1 \le i \le \delta (v)-1$, add an edge between $z^v_{u_i}$ and $x^v_{u_{i+1}}$ (see Fig. 1). This completes the construction of $F^v$. To complete the construction of $G'$, for each edge $uv \in E(G)$, add an edge between $x^u_v$ in $F^u$ and $x^v_u$ in $F^v$. The construction can clearly be carried out in polynomial time.

As above, let $v \in V(G)$ and let $\{u_1,u_2,\ldots ,u_{\delta (v)}\}$ be the set of vertices adjacent to *v*, ordered arbitrarily.

*Claim 1: In a 2-frugal 3-colouring of* $F^v$,* the vertices* $x^v_{u_i}$* for *$1 \le i \le \delta (v)$ *must receive the same colour.*

It is sufficient to show that $x^v_{u_i}$ and $x^v_{u_{i+1}}$ must receive the same colour. Let $\{1,2,3\}$ be the set of colours, and suppose $x^v_{u_i}$ has colour 1 without loss of generality. Then, $y^v_{u_i}$ has colour 2 without loss of generality, and then, the three vertices adjacent to both $y^v_{u_i}$ and $z^v_{u_i}$ must use the colour 1 exactly once, and the colour 3 exactly two times as a result of the frugality condition. Then, $z^v_{u_i}$ must receive the colour 2, and $x^v_{u_{i+1}}$ must receive the colour 1 to satisfy the frugality condition for $z^v_{u_i}$. This proves the claim.

We refer to the colour used to colour the vertices $x^v_{u_i}$ in $F^v$ as the colour of $F^v$.

*Claim 2:* $F^v$* can be coloured, such that, for* $1 \le i \le \delta (v)-1$, $z^v_{u_i}$ *and* $y^v_{u_{i+1}}$* use different colours.*

Without loss of generality, we may assume that the colour of $F^v$ is 1. The proof of the above claim shows that $y^v_{u_i}$ and $z^v_{u_i}$ have the same colour for each *i*, $1 \le i \le \delta (v)$. However, a colouring that satisfies the conditions of the claim is easily obtained by colouring $y^v_{u_i}$ and $z^v_{u_i}$ with colour 2 when *i* is even, and colour 3 when *i* is an odd. Then, since $x^v_{u_i}$ and $x^v_{u_{i+1}}$ share a colour, and $y^v_{u_i}$ and $z^v_{u_i}$ share a colour, we can always colour the three vertices of $H^v_{u_i}$ which are adjacent to both $y^v_{u_i}$ and $z^v_{u_i}$ while maintaining the frugality condition. This proves the claim.

*Claim 3: If* *u* *and* *v* *are adjacent in* *G*,* then the colour of *$F^u$ *is different than the colour of *$F^v$.

By construction, $x^u_v$ and $x^v_u$ are adjacent in $G'$, and hence must be assigned different colours in any 2-frugal 3-colouring of $G'$. By definition, the colour of $x^u_v$ is the colour of $F^u$ and the colour of $x^v_u$ is the colour of $F^v$, and therefore, the claim is proved.

Suppose a 3-colouring *c* of *G* is given. To extend this to a 2-frugal 3-colouring $c'$ of $G'$, let *c*(*v*) be the colour of $F^v$ in $c'$. By Claim 2, we can assume that our colouring of $F^v$ is such that $z^v_{u_{i}}$ and $y^v_{u_{i+1}}$ do not have the same colour, and since $z^v_{u_{i}}$ and $y^v_{u_{i+1}}$ are two of the three neighbours of $x^v_{u_{i+1}}$, the frugality condition must be satisfied for $x^v_{u_{i+1}}$. The vertices $x^v_{u_i}$ are the only vertices of $F^v$ with neighbours outside of $F^v$. Since our colouring of $F^v$ satisfies the frugality condition, our colouring $c'$ also satisfies the frugality condition. It remains to show that $c'$ is a proper colouring. Since our colouring of $F^v$ is proper for each $v \in V(G)$, this follows immediately from the fact that *c* is a proper colouring, and from Claim 3. Therefore, $c'$ is a 2-frugal 3-colouring of $G'$.

Finally, suppose a 2-frugal 3-colouring $c'$ of $G'$ is given. By Claim 1, for each $v \in V(G)$, $F^v$ has a well-defined colour. We can obtain a well-defined 3-colouring *c* of *G* from $c'$, by letting *c*(*v*) be the colour of $F^v$ in $G'$. For any edge $uv \in E(G)$, the vertex $x^u_v$ of $F^u$ and the vertex $x^v_u$ of $F^v$ are adjacent in $G'$ by construction, and therefore, $x^u_v$ and $x^v_u$ are assigned different colours by the proper colouring $c'$. Hence, $F^u$ and $F^v$ are assigned different colours by $c'$, and so, our colouring *c* will assign different colours to *u* and *v*. Therefore, *c* is a proper 3-colouring of *G*.

Therefore *P*(2, 3)-colouring is NP-complete. $\square$

Let *G* be a graph. The *corona of* *G with respect to* $K_{k-1}$ is the graph obtained from *G* by, for each vertex *v* of *G*, creating a copy of $K_{k-1}$ and adding edges, such that *v* is adjacent to each vertex of its copy of $K_{k-1}$.

### Lemma 3.2

Let $t\ge 1$ and $k\ge 1$ be integers. Then, *P*(*t*, *k*)-colouring polynomially transforms to $P(t+1, k)$-colouring.

### Proof

Let *G* be an instance of *P*(*t*, *k*)-colouring. The instance of $P(t+1, k)$-colouring obtained from *G* is the graph *H* which is the corona of *G* with respect to $K_{k-1}$. The transformation can clearly be carried out in polynomial time. We will show that *G* has a *t*-frugal *k*-colouring if and only if *H* has a $(t+1)$-frugal *k*-colouring.

For a colouring *c* of *G*, a colour *x*, and a vertex $v \in V(G)$, let $|c_x(v)|$ denote the number of times in the colouring *c* that the colour *x* appears in the neighbourhood of *v*.

Let *c* be a *t*-frugal *k*-colouring of *G*. Clearly, *c* can be extended to a proper colouring $c'$ of *H* by, for each $v \in V(G)$, colouring the copy of $K_{k-1}$ associated with *v* using each of the $k-1$ colours in $\{1,2,\ldots ,k\} - \{c(v)\}$ exactly once. Then, for each colour $x \in \{1,2,\ldots ,k\} - \{c(v)\}$, the number of times *x* appears in the neighbourhood of *v* is exactly $|c_x(v)| + 1$. Since *c* is *t*-frugal, we have $|c_x(v)| + 1 \le t + 1$. Then, since every vertex in each copy of $K_{k-1}$ is adjacent to exactly one vertex of every colour other than its own, we have that $c'$ is a $(t+1)$-frugal *k*-colouring of *H*.

Now, let $c'$ be a $(t+1)$-frugal *k*-colouring of *H*. We claim that the colouring *c* of *G* induced by $c'$ is a *t*-frugal *k*-colouring. To see this, observe that for every $v \in V(G)$ and every $x \in \{1,2,\ldots ,k\} - \{c'(v)\}$, $|c_x(v)| = |c'_x(v)| - 1$, as *x* must be used exactly once in $c'$ to colour the copy of $K_{k-1}$ in *H* associated with *v*. Since $c'$ is $(t+1)$-frugal, we have $|c_x(v)| = |c'_x(v)| - 1 \le (t+1) - 1 = t$, and therefore, *c* is a *t*-frugal *k*-colouring of *G*.

We can now prove Theorem 1.2.

### Proof (Theorem 1.2)

If $k = 1$, then a graph can be *P*(*t*, *k*)-coloured if and only if it contains no edges. If $k = 2$, then a graph can be *P*(*t*, *k*)-coloured if and only if it is bipartite with $\Delta \le t$. If $k = 3$, then for $t = 1$, the statement is clear (as discussed in the introduction), and for $t \ge 2$, the statement follows from Lemma 3.1 and induction on *t*. If $k \ge 4$, then the statement follows from the theorem of McCormick and Thomas [24] and induction on *t* using Lemma 3.2. $\square$
